# From Termination Dependent Chemical Sensitivity of Spin Orientation in All-bcc Fe/Co Magnetic Superlattices toward the Concept of an Artificial Surface of a Ferromagnet

**DOI:** 10.1021/acs.jpclett.2c02139

**Published:** 2022-09-06

**Authors:** M. Ślęzak, P. Dróżdż, K. Matlak, A. Kozioł-Rachwał, A. A. Sasikala Devi, M. Alatalo, T. Ślęzak

**Affiliations:** † Faculty of Physics and Applied Computer Science, AGH University of Science and Technology, 30-059 Kraków, Poland; ‡ National Synchrotron Radiation Centre SOLARIS, Jagiellonian University, 30-392 Kraków, Poland; § Nano and Molecular Systems Research Unit, University of Oulu, 90014 Oulu, Finland

## Abstract

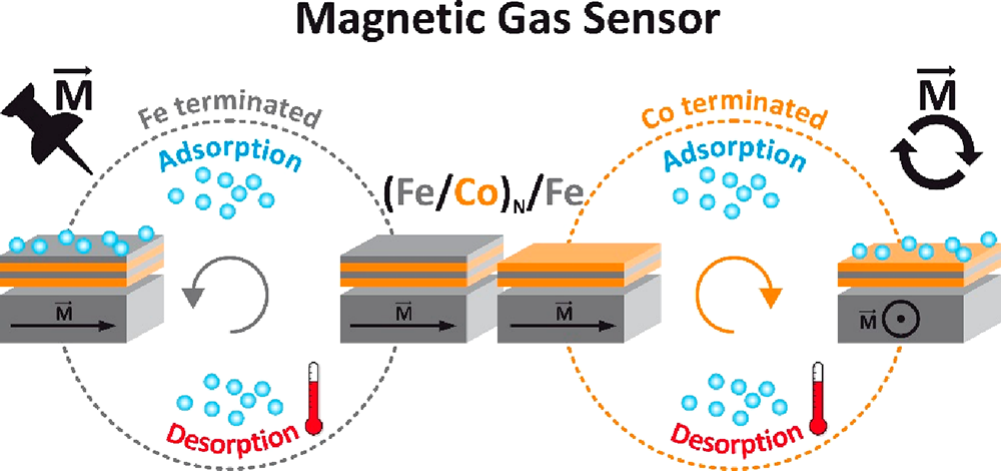

Adsorption of gases on the surface of all-bcc (Fe/Co)_N_ superlattices drives the in-plane, 90° magnetization rotation of the bulk-like Fe(110) supporting ferromagnet. Both experimental and theoretical results prove that terminating the surface of (Fe/Co)_N_ superlattices either by Co or by Fe switches “ON” or “OFF” the spin orientation sensitivity to adsorption. Results indicate that purely surface limited adsorption processes strongly modify the magnetic anisotropy of the entire (Fe/Co)_N_ superlattice, which acts as a kind of “artificial” surface of the bulky Fe(110) ferromagnet. Such an artificial magnetic surface anisotropy concept not only enhances the surface contribution in classical surface–bulk competition but also provides its additional chemical sensitivity.

Tailoring magnetic anisotropy (MA)^1^ of ultrathin ferromagnetic films has been for a long time one of the key tasks in both spintronics and fundamental research focused on magnetism of low-dimensional systems.^2,3^ From the technological point of view, since the giant magnetoresistance (GMR) discovery in 1988^4^ which has opened new possibilities for construction of the hard-drive read heads, research on surface engineering and nanomagnetism for spintronic applications has attracted a great deal of attention. The oscillatory behavior of the interlayer exchange coupling^5^ allowed to introduce the concept of synthetic magnetic free layers in the so-called magnetic tunnel junctions (MTJ) devices.^6^ Research on spin valves in ferromagnetic multilayered sandwiches with nonmagnetic metallic spacers,^7^ room temperature ∼600% tunneling magnetoresistance (TMR),^8^ perpendicular magnetic anisotropy (PMA),^9,10^ and the possibility of its voltage control^11,12^ along with the spin-transfer-torque (STT)^13^ write mechanisms and spin Hall effect (SHE)^14^ highlight the promising opportunities for future low-power and high-speed spintronic devices. Concerning surface and interface engineering of both perpendicular^15^ and in-plane^16^ magnetic anisotropy in ultrathin films and multilayers, numerous successful attempts have been reported that often relied on tuning the balance between contributions from the thickness and the temperature-dependent volume and interface MA contributions.^17−20^ Another possibility, direct modification of interface MA or interlayer magnetic coupling, has also been demonstrated by means of gas adsorption^21,22^ and absorption,^23^ respectively. In this report, we demonstrate *stricte* surface sensitive mechanisms to control the spin orientation of a bulk-like ferromagnet. In particular, our experimental and theoretical results unambiguously prove that terminating the surface of Fe/Co superlattices grown on a bulky Fe(110) film either by Co or by Fe switches “ON” or “OFF” the spin orientation sensitivity to adsorption of gases. As a result, the magnetization of the bulk-like, 500 Å-thick Fe(110) ferromagnet can be rotated by 90° within the sample plane by purely surface limited effects, namely, adsorption of residual gases on the surface of Co terminated (Fe/Co)_N_/Fe(110) multilayers. Interestingly, our findings indicate that the gas adsorption process modifies the overall magnetic anisotropy of Fe/Co superlattices and not only Co sublayers terminating the whole system. Accordingly, an (Fe/Co)_N_ stack grown on an Fe(110) film can be regarded as a chemically sensitive artificial surface of a thick Fe(110) system. The (Fe/Co)_N_ superlattice magnetic anisotropy plays a role of artificial magnetic surface anisotropy equivalent to the magnetic surface anisotropy of the pure Fe(110) film that influences the magnetization direction of the underlying Fe layer. Such magnetic surface anisotropy engineering not only enhances the surface influence on the magnetization direction of the Fe(110) film but also provides an additional degree of freedom in Fe magnetization direction control as only the Co terminated system rotates its magnetization upon the gas adsorption process, while for Fe termination almost no adsorption induced effects are found.

The heart of the system that we chose for our research consists of epitaxial mesoscopic Fe films grown on a W(110) single crystal surface. In the Fe/W(110) system,^24,25^ the evolution of the uniaxial magnetic anisotropy with the increasing thickness of the Fe layer results in the spin reorientation transition (SRT), in which Fe magnetization switches from the [11̅0] to the [001] in-plane direction.^26^ Surface magnetic anisotropy (SMA) of such uncovered Fe(110) films was in the past modified by the deposition of noble metal overlayers (Au, Ag),^27−29^ adsorption of oxygen,^28^ or ultrahigh-vacuum (UHV) residual gases.^30^ Consequently, the critical SRT thickness, which for uncovered Fe(110) films varies from 60 Å up to 130 Å depending on the preparation recipe, was drastically lowered (down to ∼10 Å) in the case of Au/Fe bilayers and moderately modified by adsorption of gases on the surface. On the other hand, using metastable bcc Co overlayers leads to a large increase of magnetic surface anisotropy and critical SRT thickness in Co(110)/Fe(110) bilayers,^31^ which suggests that the increasing number of Fe/Co interfaces^32,33^ in the system can further increase SMA and critical thickness of SRT. This can be experimentally realized by preparing epitaxial (Fe/Co)_N_ multilayers on the surface of a bulk-like Fe(110) ferromagnetic film.

The structural and magnetic properties of epitaxial (Fe/Co)_N_/Fe(110)/W(110) samples were in situ studied. The mesoscopic Fe(110) films with a thickness of several nanometers up to 50 nm were deposited using molecular beam epitaxy (MBE) on an atomically clean W(110) single crystal, at room temperature. After annealing at 675 K, high quality epitaxial Fe(110) films are in such a way prepared. Next, on the Fe(110) film, (Fe/Co)_N_ superlattices were deposited at room temperature using the special shutter placed in front of the sample. A wedge of a mesoscopic Fe(110) film is covered by (Fe/Co)_N_ superlattices with a fixed thickness of each Co and Fe sublayer. The movement of the shutter during the preparation process allowed us to produce macroscopic sample areas (stripes) with alternating Co and Fe surface terminations and increasing number of (Fe/Co) repetitions *N*.

After each processing step, the structure of the surface was in situ monitored using low-energy electron diffraction (LEED). These structural studies clearly indicate the (1 × 1) bcc structure of each Fe(110) sublayer and reconstructed, metastable bcc structure in the case of Co sublayers,^34^ almost independent of the *N* number of the Fe/Co sequence repetitions (please see Figure 2S in the Supporting Information).

The magnetic properties of the (Co/Fe)_*N*_/Fe(110) systems were studied in situ as a function of the Fe thickness, number of (Co/Fe) repetitions *N*, and sample surface termination, i.e., Co or Fe. For this purpose, a longitudinal magneto-optic Kerr effect (μMOKE) microscope, attached to our UHV system, was used.^35^ The field of view of the MOKE system was tuned to cover the whole sample area, which was 8 mm in diameter. A series of MOKE images were taken as a function of the external magnetic field *H*, which was applied along the W[11̅0] in-plane direction. Magnetic hysteresis loops could be extracted for any selected sample region of interest (ROI), which can be as small as one pixel. We followed the same methodology for MA studies as in the case of previously reported ferromagnetic^30,31^ and ferromagnetic/antiferromagnetic bilayer systems.^35,36^ For the wedged samples and those covering a wide range of *N*, the acquisition of a single MOKE “movie” as a function of the external magnetic field provided a full data set for the magnetization-reversal measurements. Moreover, all hysteresis loops were obtained under the same experimental conditions, i.e., at the same sample temperature, with the same possible sample misalignments and with the same magneto-optical artifacts, if any.

The adsorption of residual gases took place in the UHV μMOKE chamber. The typical mass spectrum in this chamber, collected using a quadrupole mass spectrometer, is presented in Figure 1S of the Supporting Information. It shows dominating the partial pressure of molecular hydrogen (H_2_) and a significant contribution from carbon monoxide (CO) as well as traces of other vacuum components like CO_2_ or H_2_O.

In Figure 1a,b, we present exemplary MOKE results for Co_5Å_/Fe_5Å_ superlattices grown on a wedged 80–300 Å Fe(110) film. Specifically, differential MOKE images of the sample surface at the remanence state are shown. To enhance the magnetic contrast, we subtracted a reference image taken at saturation in an external magnetic field along [11̅0] from the image taken at remanence. Consequently, the dark area is where the remanent magnetization remained along the saturation direction, [11̅0], whereas the bright area corresponds to the [001] magnetization direction in the remanent state.

**Figure 1:**
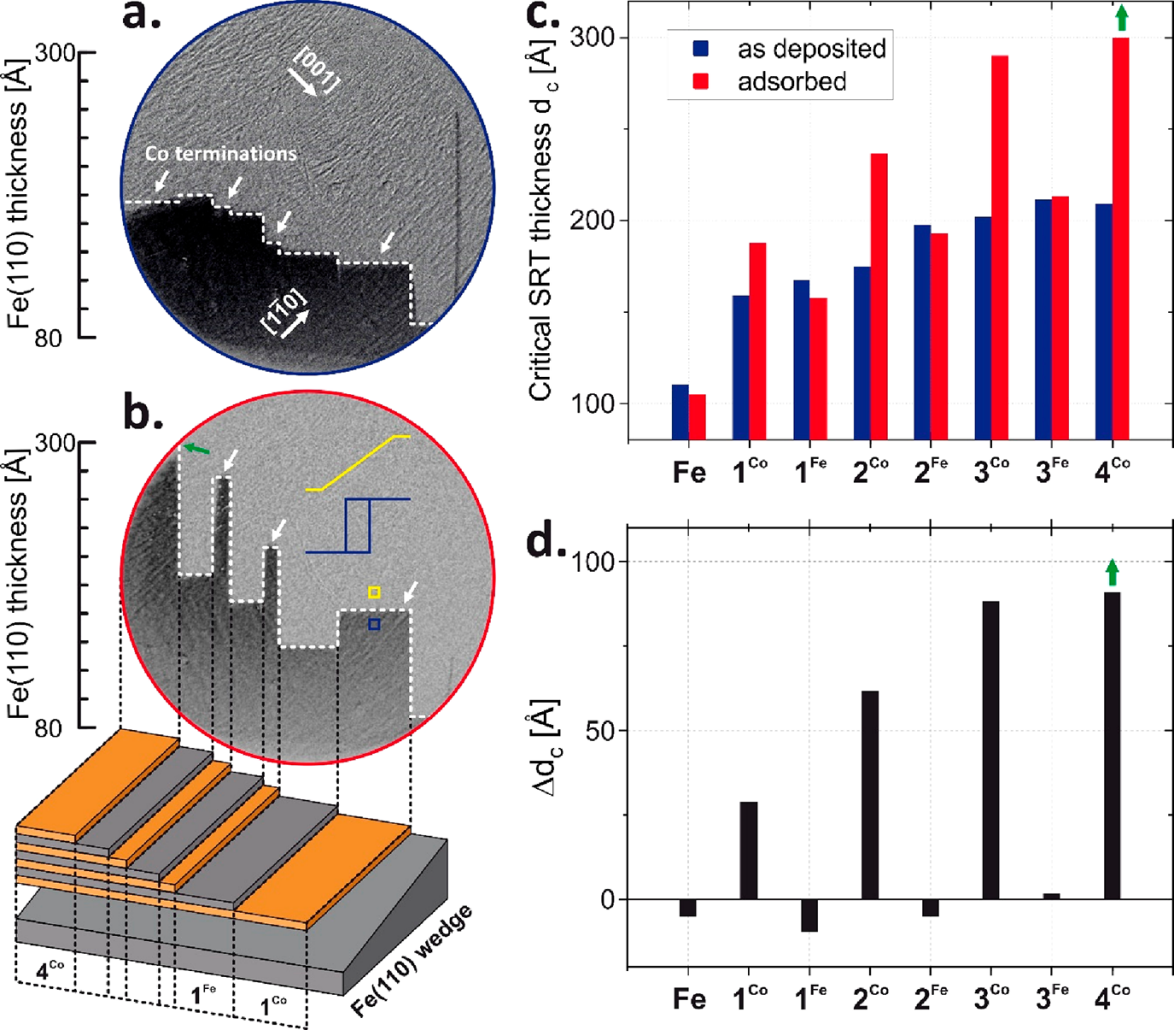
Differential MOKE images of the sample at the remanence states before (a) and after (b) the adsorption induced modification of the surface. The thickness of mesoscopic Fe(110) is continuously changing as shown in the schematic three-dimensional sketch of the sample at the bottom of (b). The dark area in the MOKE images is where the remanence magnetization remained along the saturation direction, [11̅0], whereas the bright area corresponds to the [001] magnetization direction in the remanent state. The white dotted lines mark the SRT borders between dark and bright areas depending on the N and sample termination (Fe or Co). Typical easy and hard axis loops are schematically drawn with blue and yellow colors in (b) for regions before and after SRT, respectively. Short arrows indicate Co terminated macroscopic stripes on the sample. Note that the width of each particular area is intentionally varying from stripe to stripe to allow unambiguous identification of termination and the *N* value for each sample region during the analysis process. The field of view diameter in the presented MOKE images is 8 mm. In (c) and (d) quantitative analysis of critical SRT thickness *d*_c_ (c) and its adsorption induced change Δ*d*_c_ (d) are presented.

In Figure 1a, the magnetic state of the as prepared sample is imaged, where the white dotted line marks the border between dark and bright areas that are magnetized along [11̅0] and [001] in-plane bcc directions, respectively. Analysis of magnetic hysteresis loops that can be extracted for any selected ROI confirms that the dotted line represents the critical Fe thickness *d*_c_ of the in-plane spin reorientation transition and can be therefore treated as a measure of in-plane MA strength. For clarity, in Figure 1b with blue and yellow colors we present schematically typical easy and hard axis loops characteristic for regions before and after thickness induced SRT, respectively. It has to be noted that (Co/Fe)_*N*_ and Fe(110) components are strongly exchange coupled, and consequently the overall epitaxial system behaves (for example, under application of external magnetic field) like a single, magnetically homogeneous ferromagnet. In the as prepared sample (Figure 1a), the MA is weakly dependent on the termination of the Fe/Co superlattice. After the exposure to the total ∼40 L of UHV residual gases, the MOKE image (Figure 1b) changes drastically. (For magnetic anisotropy dependence on the increasing exposure to residual gases, please see Figure 4S in the Supporting Information.) The *d*_c_ and as a result the MA strength are now strongly enhanced at Co terminated areas, while they remain almost unchanged for Fe terminations. This provides the first and very clear evidence that the adsorption of residual gases strongly modifies the in-plane magnetic anisotropy of Co terminated superlattices. Moreover, magnetization of even 30 nm thick, buried Fe(110) can be rotated by 90° within the sample plane by adsorption processes that are naturally limited to the very surface of the system. (In fact, in the Supporting Information we show that 50 nm thick Fe(110), the thickest examined layer, can be reoriented; please see Figure 5S.) In order to provide direct evidence of adsorption induced modifications of the sample surface, precise LEED studies were performed for clean and adsorbed states of the sample; please see Figure 2S and its description presented in the Supporting Information. Quantitative analysis of the presented MOKE images is shown in Figure 1c,d. Critical SRT thickness for both as deposited and adsorbed states of the sample is plotted as a function of *N* and superlattice Fe (N^Fe^) or Co (N^Co^) termination (Figure 1 c), while the adsorption induced change of the critical SRT thickness is shown in Figure 1d. It can be clearly seen that adsorption selectively and strongly enhances the magnetic surface anisotropy at Co terminated superlattices (1^Co^, 2^Co^, 3^Co^, 4^Co^). In the case of the 4^Co^ region, the critical SRT thickness *d*_c_ for the adsorbed surface cannot be precisely determined, as it is higher than 300 Å, the maximum Fe(110) thickness available in this sample. This means that adsorption increases *d*_c_ by ∼100 Å or more as schematically marked by green symbols in Figure 1d. Such an adsorption induced change of *d*_c_ and magnetic surface anisotropy is huge, especially when compared to the ∼5 Å decrease of *d*_c_ for uncovered Fe(110), as shown in Figure 1c,d. The plot of adsorption induced change of critical SRT thickness for the sample with higher numbers of Fe/Co repetitions *N* can be found in Figure 3S of the Supporting Information. Independently of technical problems with studies on samples with higher *N* (described in Supporting Information), it can be concluded that the adsorption induced enhancement of the critical SRT thickness is not saturating with increasing number of repetitions, at least up to the maximum studied *N* = 8.

From the point of view of potential applications, for example, in gas sensors, the reversibility of such adsorption induced spin reorientation is crucial. As presented in detail in the Supporting Information (Figure 2S), the adsorption of residual gases on the surface can be partially reversed by short annealing of the sample at 475 K. After the annealing induced desorption process, the sample was again readsorbed by residual gases. Corresponding “clean surface” (1)–“adsorption” (2)–“desorption” (3)–“readsorption” (4) reversibility was documented by following the magnetic properties of the system. An example is shown in Figure 2 for stripe 1^Co^, where differential MOKE images at remanence states are shown in the vicinity of the SRT. It is clear from Figure 2a that the critical SRT thickness and as a result the magnetic anisotropy are not fully reversible during a (1 −(2)–(3)–(4) cycle. However, it is possible to define the ROI (*d*_Fe_ ∼ 160 Å) for which the magnetization of the whole ferromagnetic system rotates by 90° back and forth in the reversible sequence [001]–[11̅0]–[001]–[11̅0] of orthogonal crystallographic directions within the (110) bcc plane. Corresponding magnetic hysteresis loops are plotted in Figure 2b. Clearly, for selected ROI, the magnetic hysteresis loop reversibly switches between the typical hard axis and the easy axis like one during the (1–4) cycle. This is summarized in Figure 2c, where normalized magnetization in the remanence state is presented (red circles) and compared with the LEED “fingerprint” of adsorption and desorption (black triangles), as defined in the Supporting Information. One can note a slight change of the coercivity for loops #2 and #4 and the anisotropy field value for loops #1 and #3 in Figure 2b most probably because of incomplete desorption of adsorbed atoms on the Co surface. A higher annealing temperature/time would be required to clean the surface more precisely; however, in such a case the Co/Fe multilayer structure can be destroyed (mixing at the interfaces). However, these are not changes of coercive/anisotropy fields that are the most crucial for applications; whether the magnetization reorients by 90° in adsorption–desorption cycles is important. Indeed the magnetic anisotropy modification is only partially reversible, but the 90° switching of magnetization can be performed multiple times; therefore, such an idea may be used for application in real devices, where, for example, the anisotropic magnetoresistance can be adopted to electrically detect the spin state of the system.

**Figure 2:**
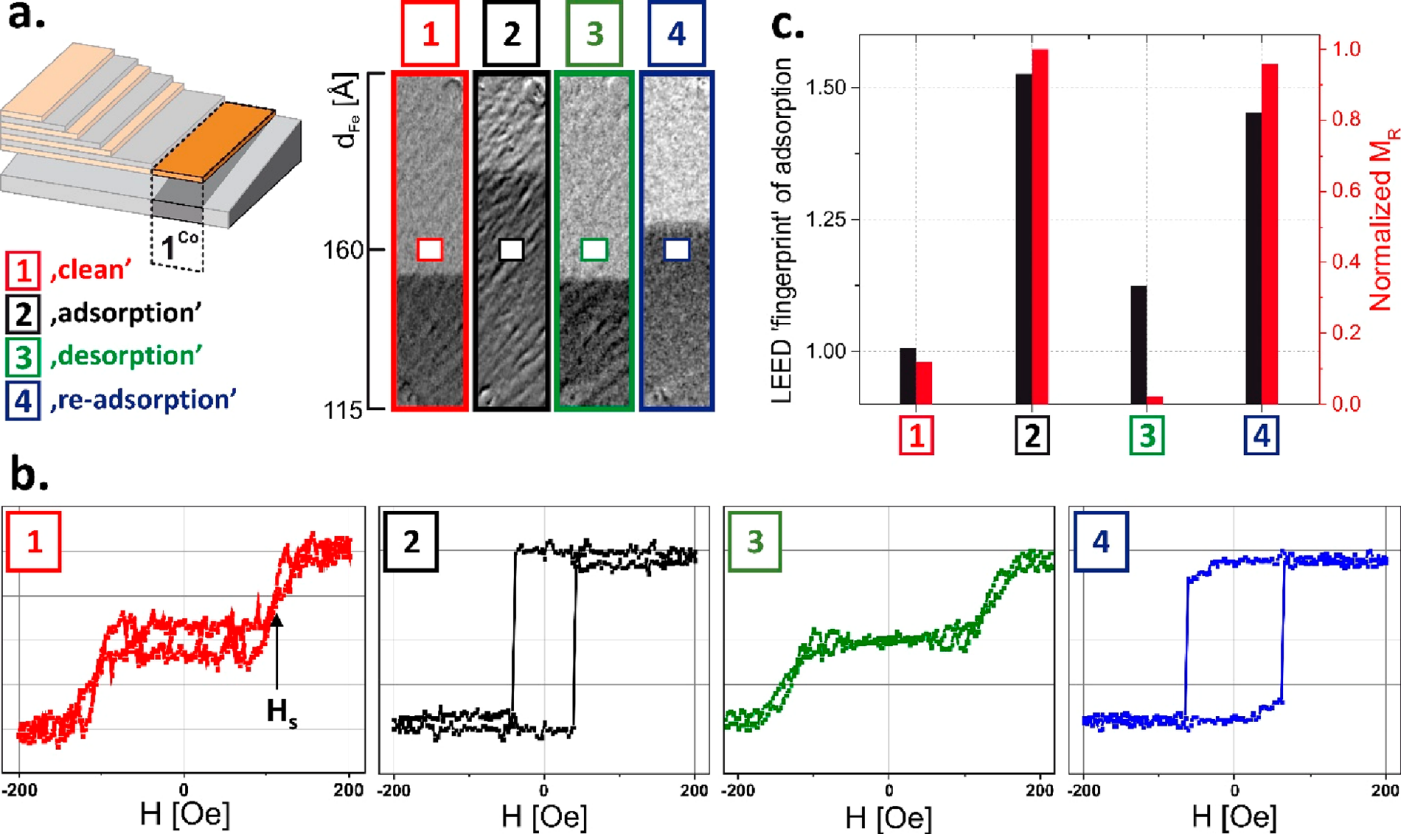
(a) Differential MOKE images at the remanence state, in the vicinity of the SRT in stripe 1^Co^, as acquired for the (1–4) states of the sample. (b) Corresponding magnetic hysteresis loops are presented showing the reversible 90° switching of the in-plane easy axis for selected ROI. (c) Plots of the (1–4) cycle for (red) normalized magnetization in the remanence state as determined from loops presented in (b) together with a (black) LEED “fingerprint” of adsorption and desorption, as defined in the Supporting Information.

To get further insight into the experimental findings, density functional theory (DFT) based simulations were conducted using the plane wave based code, Vienna Ab-Initio Simulation Package VASP.^37−41^ For corresponding computational details, we refer to the Supporting Information. As a target of our theoretical considerations, we choose the most interesting case of two atomic layers (AL) of Fe and Co, forming the Fe(2 AL)/Co(2 AL) bi- or multilayer. (Please note that, from the calculations point of view, bilayers and multilayers with a higher number of Fe/Co repetitions are equivalent and represented by periodically repeating UHV/Fe/Co/UHV regions. For the same reason, the Co and Fe terminations can be distinguished only by attaching adsorbate atoms or molecules to the chosen termination.) The atomic magnetic moments were oriented along each of the two in-plane directions of interest, namely, *Y* ([001]) and *X* ([11̅0]). The total energy difference between these two orientations was next calculated and defined as the magnetic anisotropy energy (MAE = *E*_*X*_ – *E*_*Y*_).^33^ From this definition, a negative MAE implies an easy axis along the *X* direction and positive MAE indicates that the *Y* axis is the easy axis. Table 1 presents the results of MAE calculations for (i) an UHV surrounded bilayer and in the case of (ii) H atoms or (iii) CO molecules attached either to Co or to an Fe terminated bilayer surface. First of all, one can note that magnetic anisotropy prefers an in-plane *X*([11̅0]) direction (negative MAE) for both clean (interface with UHV) and adsorbed surfaces, independently of the superlattice surface termination in agreement with our experimental observations. MOKE results clearly show that, independently of the number of repetitions, surface termination, or state (clean or adsorbed), Fe/Co superlattices enhance the [11̅0] magnetic anisotropy of the system, as compared to uncovered Fe(110). This can be clearly seen, for example, by the increase of critical SRT thickness in Figure 1c. What is more important is that the results presented in Table 1 confirm the adsorption induced mechanism of the magnetic anisotropy enhancement and its surface termination sensitivity. In particular, attaching the H atoms or CO molecules to the Fe terminated surface of the Fe/Co bilayer results either in a minor change of MAE (H atoms) or even in a significant decrease (CO molecules) of the intrinsic [11̅0] anisotropy of the Fe/Co bilayer. In contrast, and in good agreement with experiment, in the case of Co termination, calculations confirm the adsorption induced [11̅0] magnetic anisotropy as the values of MAE become more negative with respect to the clean bilayer surface.

**Table 1:** Magnetic Anisotropy Energy (MAE) of the Fe(2 AL)/Co(2 AL) Bilayer Calculated for (i) UHV Surrounded Bilayer and in the Case of (ii) H Atoms or (iii) CO Molecules Attached either to the Co or to the Fe Terminated Bilayer Surface^a^

	UHV	H/Fe	H/Co	CO/Fe	CO/Co
MAE [meV]	–0.76	–0.65	–1.10	–0.50	–0.90
enhanced/suppressed by		–14%	+45%	–34%	+18%

a Symbols + and – in the third row of the table correspond to enhanced (+) or suppressed (−) [11̅0] in-plane magnetic anisotropy induced by H atoms or CO molecules, with respect to the UHV/bilayer interface.

Similarly to the recent report by Chen et al.,^42^ dissociative adsorption of hydrogen was considered as H_2_ molecules were found to form very weak bonding with both Fe and Co surfaces. This is also in agreement with calculations concerning H_2_ adsorption on Fe(110),^43^ where stable Fe–H bonds were concluded as the final state after the H_2_ molecular adsorption and subsequent dissociation. With this consideration, results presented in Table 1 stay also in a qualitative agreement with the partial reversibility of adsorption–desorption induced magnetic anisotropy modifications, as presented in Figure 2. Higher annealing temperatures would be necessary to break the stable H–Fe bonds and make the reversibility of magnetic switching complete.

To provide the deeper insight into the chemistry of the adsorption induced magnetic anisotropy, the orbital and atom resolved density of states (DOS) including spin polarization was calculated for the pristine Fe/Co bilayers and also with H adsorbed on the Co and Fe terminations. The results of these calculations are shown in Figures 7S (Fe and Co d orbitals) and 8S (hydrogen orbitals) of the Supporting Information. The d-band center was calculated for the pristine and H adsorbed bilayers to understand the relationship between the change in the electronic structure of the surface upon adsorption. Hammer and Nørskov^44^ have shown that the adsorption of gaseous atoms on the transition metal surfaces changes the d band center, and this in turn affects the strength of the interaction. We find that the shift in the d band center when H is adsorbed on the Fe and Co atoms is slightly different from that of the pristine bilayer. The average value is slightly shifted toward the Fermi level when H is adsorbed on the Co atom, while it is shifted away from the Fermi level when H is adsorbed on the Fe atom. It can be seen that the d band spin splitting is increased when the H atom is adsorbed on the Co atom, which in turn reduces the average value of d band center. The Bader charge analysis^45^ was carried out to understand the charge transfer between the surfaces and the adsorbed H atoms. The Bader charges are calculated for Co–H and Fe–H bonded atoms and also for the nearest neighbors to these atoms. From the Bader charges it can be seen that both Co and Fe atoms that are directly bonded to the H atom lose charge and the H atom gains charge. The charge gain is stronger for the H atom when it is attached to the Co atoms. The charge transfer results from hybridization^10,46^ of adsorbed and transition metal atoms and in turn affects the magnetic properties of Fe/Co bilayers. As a result the magnetic anisotropy energy is different for Fe and Co terminated surfaces. There is also a redistribution of charge occurring between the Fe and the Co atoms at the surface because of the adsorption of H, which to some extent explains why purely surface limited adsorption effects modify the magnetic properties of buried Fe/Co sublayers.

Finally, the dependence of the adsorption induced change of the critical SRT thickness on the repetition number *N* for the Co terminated (Fe/Co)_N_/Fe(110) system must be discussed. It is evident from Figure 1d that the gas adsorption process shifts the critical SRT thickness by about 2.9 nm for *N* = 1 while for the *N* = 2 and *N* = 3 the Δ*d*_c_ amounts to 6.2 and 8.9 nm, respectively. Obviously the gas adsorption process is localized strictly at the system surface, meaning that only the single, topmost Co sublayer from the (Fe/Co)_N_ stack is exposed to gases, which should lead to the constant (independent of the repetition number) SRT shift upon adsorption. Our results show that this clearly is not the case as almost linear evolution of Δ*d*_c_ with increasing *N* is observed for the Co terminated system. This means that the modification of the topmost Co sublayer magnetic anisotropy induced by the adsorbed gas results in similar changes of magnetic anisotropy in buried Co sublayers as the Δ*d*_c_ shift scales with the repetition number with the rate of about 3 nm/repetition. One can conclude that the adsorption of gas naturally limited to the topmost Co layer induces modification of the magnetic anisotropy equivalent to the unreal scenario of the residual gases adsorbed on all Co sublayers. In principle, such a scenario could be even potentially real and in such a case should be rather referred to as an absorption instead of adsorption of gases, in analogy to results presented by Hsu et al.^23^ Please see the section “Alternative interpretations of magnetic anisotropy modification in (Fe/Co)_N_/Fe(110)” in the Supporting Information, where such an interpretation is excluded for the present case. Our observations lead to the conclusion that (Fe/Co)_*N*_ stacks can be treated for all *N* as the artificial magnetic surface of the underlying Fe film, and their magnetic anisotropy plays the role of (artificial) magnetic surface anisotropy even though the surface of the topmost Co sublayer terminates the system structure. Taking all the above into account, the (Fe/Co)_*N*_/Fe(110) system behaves like a pure Fe(110) film with the surface represented by a (Fe/Co)_*N*_ superlattice system with a repetition number enhanced chemical sensitivity that additionally can be switched “OFF” or “ON” depending on the surface termination (Fe or Co). The origin of such a collective response of Co sublayers in a (Fe/Co)_*N*_ superlattice system to gas adsorption on the topmost Co sublayer is not clear and will be studied elsewhere, but possible spin dependent quantum well states formed within the (Fe/Co)_*N*_ superlattice for the Co terminated stacks can be taken into account. In such a scenario, the modification of magnetic anisotropy of the topmost Co sublayer observed in our experiment and confirmed by the DFT calculation can disturb such quantum well state, and hence the magnetic anisotropy of all Co sublayers is modified.

To conclude, we showed that the magnetization of the bulk-like Fe(110) ferromagnet can be rotated by 90° within the sample plane by purely surface limited effects, namely, adsorption of residual gases on the surface of Co terminated (Fe/Co)_*N*_/Fe(110) multilayers. Our MOKE and theoretical DFT results unambiguously prove that terminating the surface of the Fe/Co superlattices grown on a bulky Fe(110) film either by Co or by Fe, switches “ON” or “OFF” the spin orientation sensitivity to adsorption of gases. Moreover, the gas adsorption process modifies the overall magnetic anisotropy of (Fe/Co)_*N*_ superlattices and not only Co sublayers terminating the whole system. We propose the concept of artificial surface magnetic anisotropy in our system represented by magnetic anisotropy of the (Fe/Co)_*N*_ stack that influences the magnetization direction of the underlying Fe layer. Such magnetic surface anisotropy engineering not only enhances the surface influence on magnetization direction of the Fe(110) film but also provides an additional degree of freedom in Fe magnetization orientation control as only the Co terminated system rotates its magnetization upon the gas adsorption process, while for Fe termination almost no adsorption induced effects are found. We believe that our findings can be important for the fundamental problem of spin orientation control but also from the point of view of applications such as magnetic gas sensors.

## Supporting Information Available

Additional experimental and computational details and methods, including structural and magnetic characterization (PDF)
