# Supplementary material for: From Termination Dependent Chemical Sensitivity of Spin Orientation in All-bcc Fe/Co Magnetic Superlattices toward the Concept of an Artificial Surface of a Ferromagnet
Source: J Phys Chem Lett. 2022 Sep 6;13(36):8522–8. doi: 10.1021/acs.jpclett.2c02139 (PMC9486937; doi:10.1021/acs.jpclett.2c02139)
Supplement: Supplementary file 1 — jz2c02139_si_001.pdf [file jz2c02139_si_001.pdf]

Supplemental material for “From Termination Dependent Chemical Sensitivity of Spin Orientation in All-bcc Fe/Co Magnetic Superlattices toward the Concept of an Artificial Surface of a Ferromagnet”

M. Ślęzak[1], P. Dróżdż[1], K. Matlak[2], A. Kozioł-Rachwał[1], A. A. Sasikala Devi[3], M. Alatalo[3], T. Ślęzak[1]

[1]AGH University of Science and Technology, Faculty of Physics and Applied Computer Science, 30-059 Kraków, Poland

[2] National Synchrotron Radiation Centre SOLARIS, Jagiellonian University, 30-392 Kraków, Poland

[3] Nano and Molecular Systems Research Unit, University of Oulu, 90014 Oulu, Finland

Residual gases in UHV µMOKE chamber

The adsorption of residual gases took place in the UHV µMOKE chamber. The typical mass spectrum in this chamber, collected using quadrupole mass spectrometer is presented in Fig.1S. It shows dominating partial pressure of molecular hydrogen (H_2), significant contribution from carbon monoxide (CO) as well as traces of other vacuum components like H_2O (mass number 18) or CO_2 (mass number 44).

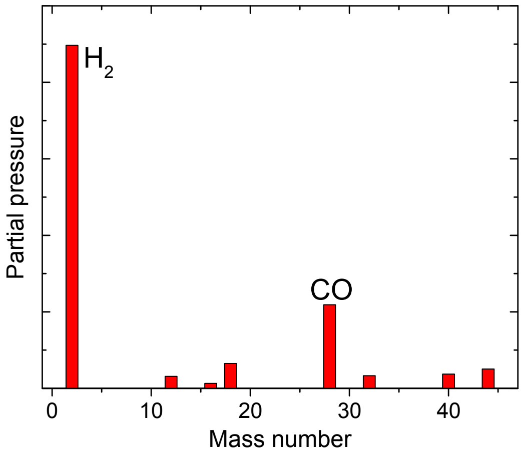

Fig. 1S. The typical mass spectrum as acquired by quadrupole mass spectrometer in UHV µMOKE chamber.

LEED study of adsorption on $(Fe/Co)_N/Fe(110)$

Adsorption induced modification of magnetic anisotropy at Co terminated superlattices is the only possible explanation for such drastic changes of magnetic properties of the overall epitaxial system. Nevertheless, in order to provide direct evidence of such adsorption driven processes we have performed detailed, stricte surface sensitive LEED studies of the sample (1) before and (2) after the adsorption as well as after additional annealing (3) (partially restoring the uncontaminated surface) of the sample and subsequent re-adsorption (4) of residual gases on the surface. Results of these studies are presented in Fig. 2S. As exemplified by the LEED pattern collected on stripe 1^{Fe}, its symmetry corresponds to unreconstructed 1x1 Fe(110) surface. This conclusion is valid for all Fe terminated superlattice stripes, independently of N. On the other hand, the LEED pattern acquired on stripe 2^{Co} (and also on all other Co terminated stripes) displays characteristic splitting of (10) diffraction spots, as seen in Fig. 2S a. This splitting is a beginning of further Co-thickness dependent evolution of characteristic superstructure that appears with increasing Co thickness. Such superstructure is a result of the periodic lattice distortion existing in two equivalent (3 × 1) reconstruction domains. Following the careful analysis of Fölsch et al. [23] and our previous report [20] we conclude that our LEED patterns on Co terminated superlattices

correspond to the metastable bcc Co(110) structure; however, the reconstructed film surface appears beyond the second Co monolayer. Characteristic superstructure observed in diffraction patterns on Co terminated superlattices makes the intensity of the additional spot (marked by red arrow in LEED spot profile in Fig. 2S a) a kind of element sensitive probe of the Co structure and allows to follow its dependence on the adsorption and desorption processes. Figure 2S b presents the intensity of the additional LEED spot as a function of the position on the sample, the same one which was used for MOKE studies presented in Fig.1 of the main article. The vertical white and grey stripes visible in the background of the Fig. 2S b mark the sample areas corresponding to subsequent Fe and Co terminated stripes respectively, directly as determined from MOKE images in Fig.1.

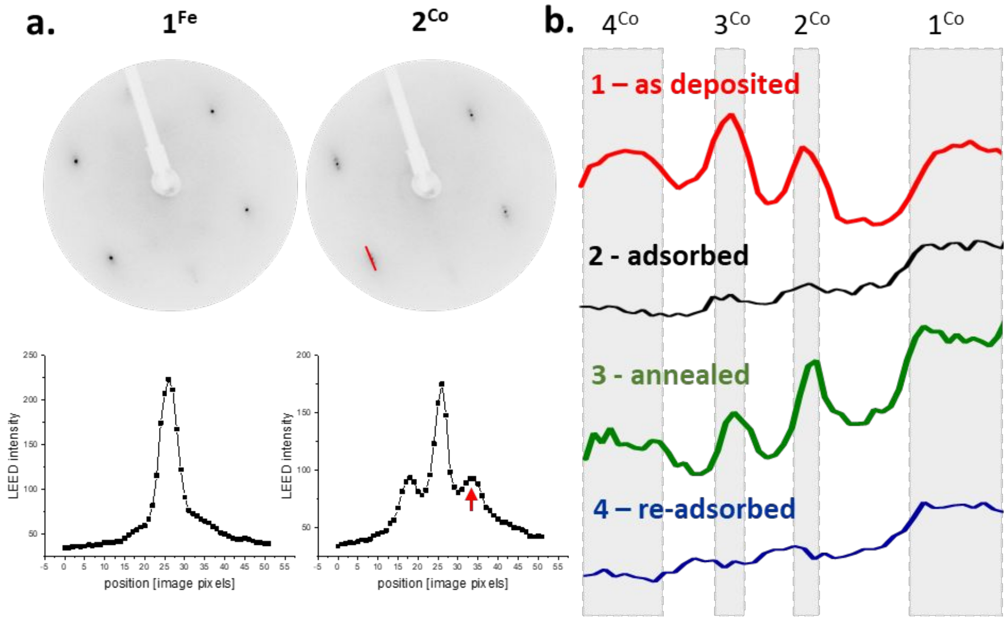

Fig. 2S. (a) LEED patterns (E = 120 eV) collected on stripes 1^{Fe} and 2^{Co} together with the corresponding intensity profile in the vicinity of (10) diffraction spot, as determined along the red line. Red arrow marks the intensity of the additional diffraction spot characteristic for Co terminated superlattices. (b) Scans of additional diffraction spot intensity vs position on the sample, as determined from LEED patterns collected in four states of the sample.

Additional diffraction spot intensity was followed at four states of the sample, namely for freshly deposited sample (1), after the surface was adsorbed with residual UHV gases (2), after restoring the surface purity by means of short annealing of the sample at 475 K (3) and again after the surface became re-adsorbed by residual gases (4). States (1) and (2) directly correspond to a magnetic state of the sample as presented in Fig.1 a and b, respectively. It can be clearly seen from Fig. 2S b that the intensity of the additional diffraction spot is enhanced at all Co terminated areas (stripes 1^{Co}, 2^{Co}, 3^{Co} and 4^{Co}) for states (1) and (3) that correspond to almost perfectly clean (1) and partially cleaned (3) surface of the as deposited and annealed sample, respectively. On the other hand, at the (2) and (4) states adsorption processes almost fully suppress the intensity of the additional spot and make the presented intensity profiles almost insensitive to Co and Fe terminations.

Alternative interpretations of magnetic anisotropy modification in $(Fe/Co)_N/Fe(110)$

The large adsorption induced change of critical thickness for increasing N is, although desired, surprising. In the following we present one possible explanation of this effect related to the sample overall structure and we show why such scenario can be excluded. Let's focus for example on 4^{Co} area in Fig.1 of the main article. At this sample area, the 3^{Co}, 2^{Co} and 1^{Co} sublayers (bot not areas) are buried. All these sublayers become surface layers on 3^{Co}, 2^{Co} and 1^{Co} areas of the sample which means that on

some areas of the sample they are fully exposed to residual gases. Assuming the lateral transfer of magnetic anisotropy one could explain in this way non-saturating critical thickness dependence on N. However, if this is the case one should also observe large, adsorption induced change of magnetic anisotropy on all Fe terminated areas, for example 3^{Fe} area in Fig.1 should exhibit critical thickness change comparable with that of 4^{Co} or 3^{Co}. This is clearly not the case (Fig.1d) as the 3^{Fe} area is totally insensitive to adsorption. Moreover, if any changes are induced on Fe terminated areas they are opposite (decrease of critical Fe thickness) to those for Co terminated areas, result which is very similar to single Fe(110) film surface. Additionally, our results are almost independent on the macroscopic areas of particular stripes as the latter were intentionally varied across the sample presented in Fig. 1 and other samples, including homogenous, Co terminated superlattices on wedged Fe(110).

In a similar way another possible interpretation can be in our opinion excluded, namely diffusion of gases along the sample normal towards buried Co sublayers. Both theoretical (Table 1) and experimental results (mass spectrum from MOKE UHV chamber and reversibility of the main effect) indicate hydrogen being responsible for the modification of magnetic anisotropy. Having in mind high mobility of hydrogen one should consider absorption rather than adsorption induced change of magnetization state in the system. Again, magnetic anisotropy on Fe terminated areas is almost unchanged during the μMOKE experiments therefore absorption processes can be ruled out. Diffusion of hydrogen into buried Co sublayers on Fe terminated areas would *directly* modify the magnetic anisotropy of all Co sublayers and should result in significant change of critical thickness also in these sample regions, which clearly is not the case in Fig. 1d.

Adsorption induced magnetic anisotropy in $(Fe/Co)_N/Fe(110)$ for sample with higher N

In Fig. 3S we present MOKE results for a sample with higher number of Fe/Co repetitions, specifically with N = 8 Co sublayers. In general, these results confirm the conclusion that the adsorption induced enhancement of critical SRT thickness is restricted to the Co terminated sample areas and is not saturating with increasing number of repetitions. One can also note significantly smaller absolute Δd_c values in Fig. 3S as compared to Fig. 1d in the body of the article. This results from technical issues related to the preparation protocol of high N samples. As the overall time for $(Fe/Co)_8$ superlattice preparation (but also for its structural characterization) was more than twice longer as compared to the sample reported in Fig.1, the adsorption sensitive Co areas were much more exposed to residual gases already before the in-situ MOKE experiment. For this reason, the "clean" state of the sample was to some extent already influenced by adsorption and thus the presented $\Delta d_c = d_{c\ adsorbed} - d_{c\ clean}$ values are smaller.

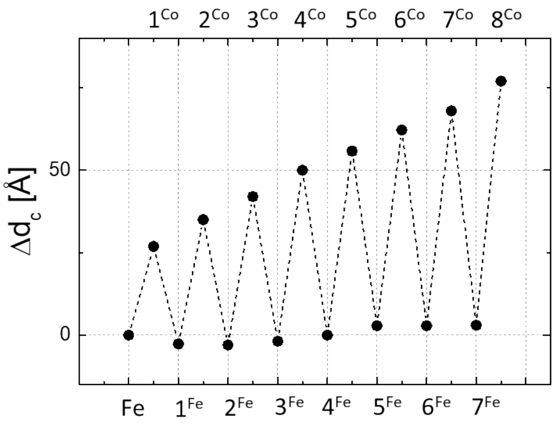

Fig. 3S. Plot of adsorption induced change of critical SRT thickness for sample with higher numbers of Fe/Co repetitions N.

In Fig. 4S we present results of more systematic study of the critical SRT thickness dependence on the exposure to residual gases. The total exposure was calculated as a product of time of the experiment and total pressure in the MOKE UHV chamber. The latter can be assumed to be almost time independent during MOKE measurements and was equal to ~ $1*10^{-9}$ Torr. Clearly, above 40 L exposure the effect starts to saturate and no further adsorption induced increase of magnetic anisotropy is observed.

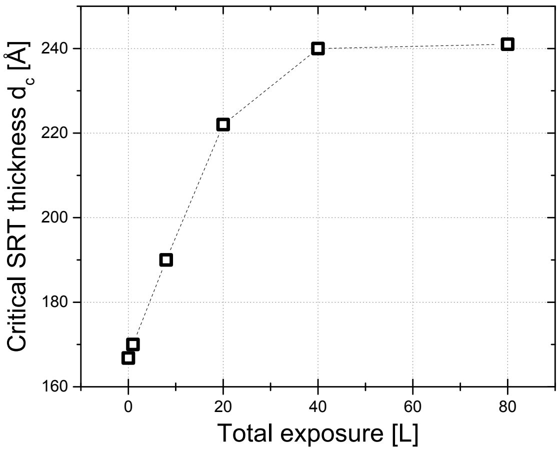

Fig. 4S. Critical thickness of SRT dependence on the exposure to residual gases. Data collected on the dedicated sample for stripe 2^{Co}.

Adsorption induced magnetic anisotropy in $(Fe/Co)_N$/Fe(110): dependence on Co thickness

Samples with wedged base Fe layer and spatially varying number of $(Fe/Co)_N$ repetitions allow for the most convenient studies of MA, as simply the critical SRT thickness is followed. However in such case one has to focus on specific thicknesses of Fe and Co sublayers in $(Fe/Co)_N$ stacks. In order to get insight into the adsorption induced MA dependence on the Co sublayer thickness, the dedicated sample was prepared, where the thicknesses of the Fe(110) base layer and superlattice Fe sublayers were fixed and equal to 500 Å and 5 Å, respectively, while each Co sublayer was wedged and covered the thickness range 0-15 Å, please see schematic drawing of the sample in Fig. 5S a. The following approach was used. Looking on available up to now literature data, 500 Å-thick Fe(110) film is much above the critical thickness of SRT, independently of type of the surface modifications or coverage used (hydrogen, carbon monoxide, oxygen, oxidation, non-magnetic or magnetic overlayers). Consequently one expects that the easy axis of such Fe(110) layer is bulk-like, i.e. along the Fe[001] direction and that it will not change even upon covering by superlattices. Therefore, by applying the in plane external magnetic field along Fe[$1\bar{1}0$] direction, the typical hard axis hysteresis loops, like those presented for sample states (1) and (3) in Fig. 2, can be locally measured. Such loops exhibit a characteristic jump of magnetization at the switching field H_s which is a good measure of the uniaxial anisotropy. In this way the in plane uniaxial magnetic anisotropy can be characterized quantitatively from single-loop measurements across the two-dimensional (d_{Co}, N) space. In order to determine the full dependence of H_s in (d_{Co}, N) space, the sample area was divided into a (50 x 50) matrix of ROIs. For each ROI a hysteresis loop was extracted by analysis of a series of MOKE images taken as a function of the external magnetic field. Consequently, H_s and in plane magnetic anisotropy, can be analyzed for any combination of Co thickness and repetition number N (or surface termination type). The size of a single ROI was 80×120 μm^2, which corresponds to the averaging of magnetic properties over a finite thickness interval Δd_{Co} = ~ 0.3 Å for each Co stripe. Stripes with varying N have 600 µm width which corresponds to a set of 5 nominally equivalent ROIs for each given N value and stripe.

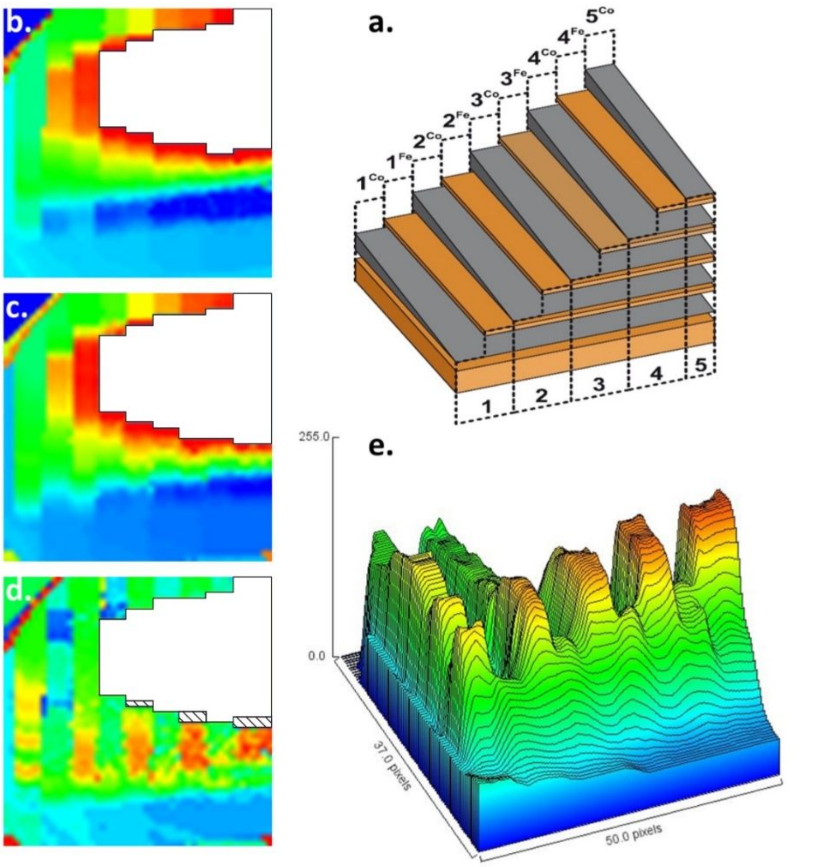

Fig. 5S. (a) Schematic drawing of the sample used to follow the two-dimensional MA maps $H_s(d_{Co}, N)$ for (b) freshly deposited and (c) adsorbed sample. In (d) adsorption induced, differential $\Delta H_s(d_{Co}, N)$ map is shown, while its corresponding three-dimensional view is presented in (e).

Results of the hysteresis curves analysis are presented in Fig. 5S b-d, where two-dimensional maps of H_s are shown for the selected sample area. Fig. 5S b presents H_s map for the freshly deposited sample. Surprisingly, regions with N and d_{Co} higher than 2.0 (2^{Fe}) and ~2 ML, respectively, are characterized by typical easy axis square hysteresis loops. It means that in these areas (marked in Fig.5S b, c with white filling) the whole ferromagnetic system is magnetized along direction orthogonal to bulk-like easy axis of bcc Fe or in other words the critical SRT thickness of the Fe(110) sublayer is above 500 Å. These regions are excluded from quantitative analysis as the induced by superlattices $[1\bar{1}0]$ – type magnetic anisotropy is too strong. In the surrounding areas the value of the switching field H_s and corresponding strength of the uniaxial anisotropy is locally visualized by the color scale. At first glance, one may judge that the $H_s(d_{Co}, N)$ maps determined for the as-deposited (Fig. 5S b) and adsorbed (Fig. 5S c) states of the sample are very similar. The red areas correspond to hard axis hysteresis loops with a small switching field (strong $[1\bar{1}0]$ anisotropy), while the green areas are characterized by the highest H_s values (weak $[1\bar{1}0]$ anisotropy and dominating bulk-like anisotropy). The large difference between the as deposited and adsorbed states of the sample becomes clearly visible in the differential ΔH_s map shown in Fig. 5S d and visualized in a three-dimensional view in Fig. 5S e. There can be no doubt that, depending on the Co thickness, adsorption induces large $[1\bar{1}0]$ anisotropy mainly in the Co terminated stripes, as reflected by orange and red areas in differential anisotropy map in Fig. 5S d or huge mountain ridges in Fig. 5S e. One can also note that selected areas, initially bulk-like magnetized along [001] direction became magnetized along orthogonal $[1\bar{1}0]$ direction after adsorption. These regions are in Fig. 5S d marked by dashed rectangles. From Fig. 5S d and e it becomes also evident that precise tuning of the Co sublayers thicknesses is crucial for magnetic anisotropy engineering and provides additional degrees of freedom in increasing the functionality of the system. It is especially well seen for 1^{Co} stripe where three distinct

maxima and oscillations of magnetic anisotropy are observed within narrow range of Co thickness. Magnetic anisotropy profiles of stripe 1^{Co} are shown in Fig. 6S a, as determined from the data presented in Fig. 5S b-d. Local minima of H_s (maximum of $[1\bar{1}0]$ anisotropy) are clearly seen in Fig. 6S a for each completed atomic layer of Co in the as deposited state of the sample, in agreement with our previous report [20]. Adsorption shifts the positions of these minima and as a result the adsorption induced modification of the magnetic anisotropy presented in Fig. 6S b displays distinct oscillations as a function of Co thickness. For Co terminated superlattices with higher N values, d_{Co} corresponding to ~ 2 atomic layers guarantees optimal (highest) adsorption induced effects. Exemplary H_s vs. N dependencies, as determined from data presented in Fig. 5S b-d for fixed Co coverage equal to 2 ML, are shown in Fig. 6S c. Corresponding adsorption induced ΔH_s dependence on the termination and repetition number is presented in Fig. 6S d. Again, the sensitivity to adsorption on Co terminated areas is well seen.

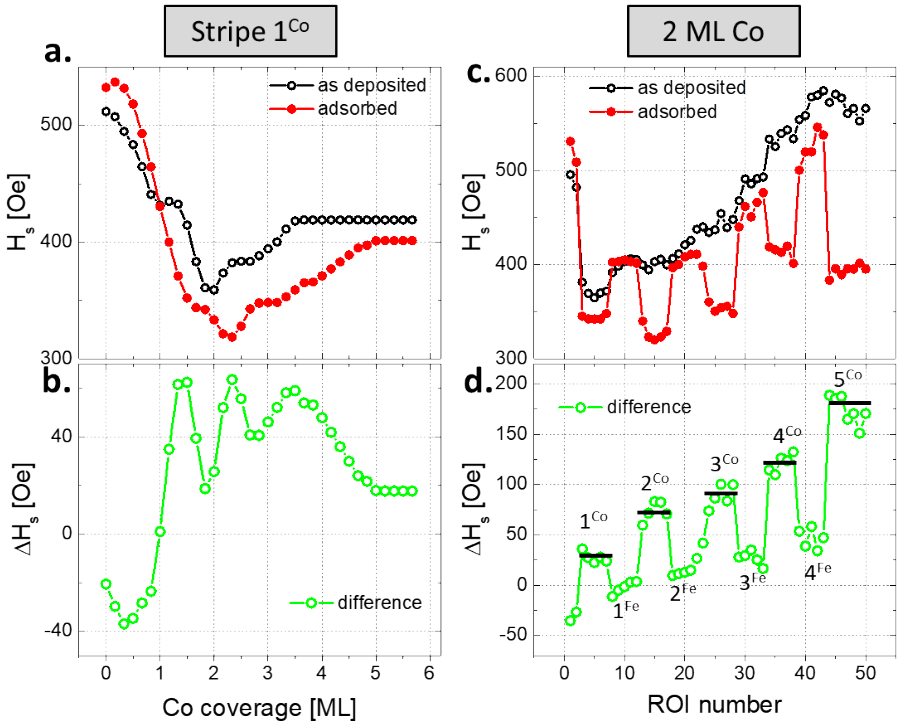

Fig. 6S. (a) Magnetic anisotropy profiles $H_s(d_{Co})$ of stripe 1^{Co} as determined from the data presented in Fig. 5S b-d, in (b) the corresponding adsorption induced $\Delta H_s(d_{Co})$ dependence is shown. (c) Exemplary H_s vs. N dependencies for fixed Co coverage equal to 2 ML with the corresponding adsorption induced ΔH_s dependence on the termination and repetition number (d).

Computational methodology

To get further insight into the experimental findings, density functional theory (DFT) based simulations were conducted using the plane-wave based code, Vienna Ab-Initio Simulation Package VASP [26-28]. The pseudopotentials were described using the projector augmented wave (PAW) method [29]. The plane wave kinetic energy cut off was fixed at 520 eV. The energy and force tolerance criteria for the relaxation were set at 1E-07 and -1E-03 respectively. The generalized gradient approximation (GGA) was used to describe the exchange and correlation interactions [30]. The Fe-Co bilayers oriented along bcc [110] direction were constructed using the supercell approach and optimization is performed using the conjugate gradient method, including spin polarization. Initially a bulk supercell was formed with 2 Fe and 2 Co layers and subjected to full structural relaxation. Further, the Fe/Co bilayer was constructed by adding a vacuum region of thickness 17 Å along the Z direction and ionic optimization is performed. The addition of vacuum layers is to minimize the interaction between periodically repeating images while infinite periodicity is maintained along X and Y directions. The optimized lattice parameters of

the Fe-Co bilayer are a= 8.012 Å, b = 2.866 Å and c = 23.012 Å, respectively. A Monkhorst-Pack K grid of 3x12x1 was used for the Brillouin zone integration. Initially self-consistent collinear calculations were performed, and the resulted ground state was used to initialize non-collinear calculations including spin-orbit coupling. During the non-collinear step, the atomic magnetic moments were oriented along the two in plane directions such as Y ([001]) and X ([1-10]). The total energy difference between these two orientations was calculated as the magnetic anisotropy energy (MAE = Ex – Ey) [22]. From this definition, a negative MAE implies an easy axis along X direction and positive MAE indicates that the Y axis is the easy axis.

Density of states of Fe/Co bilayers

The orbital and atom resolved density of states (DOS) including spin polarization was calculated for the pristine Fe/Co bilayers and also with H adsorbed on the Co and Fe terminations. In Fig. 7S we present the calculated DOS for clean (top-most panel) and H adsorbed Fe/Co bilayer in case when H atoms are attached to Fe (central panel) and Co (bottom panel) terminated bilayer surfaces. It can be seen that the bilayers are metallic with significant DOS at the Fermi level and contributed by the d-bands of Fe and Co.

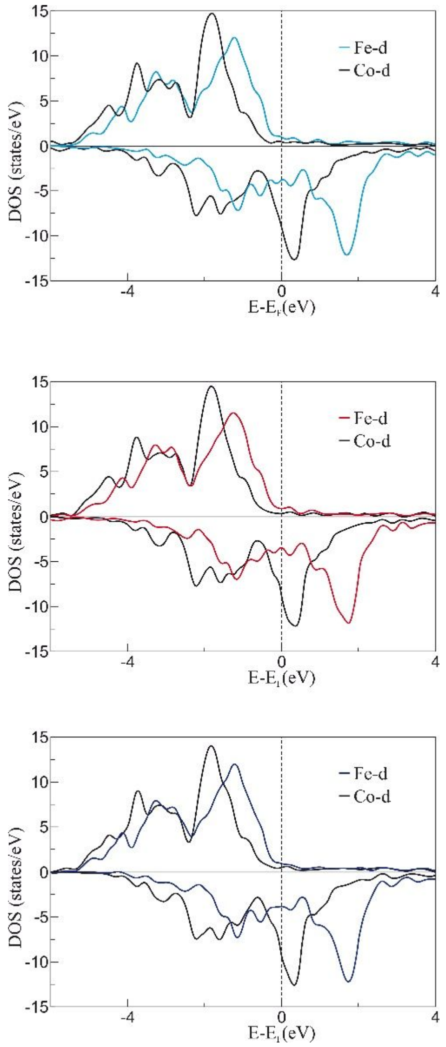

Fig. 7S. Density of states (DOS) for clean (top-most panel) and H adsorbed Fe/Co bilayer in case when H atoms are attached to Fe (central panel) and Co (bottom panel) terminated bilayer surfaces.

Further, the d-band center was calculated for the pristine and H adsorbed bilayers to understand the relation between the change in electronic structure of the surface upon adsorption. Hammer and Norskov

[44], have shown that the adsorption of gaseous atoms on the transition metal surfaces changes the d band center and this in turn affects the strength of interaction. The d-band-center is calculated with reference to the Fermi level, i.e. $E_F = 0$ eV. We find that the shift in d band center when H is adsorbed on the Fe and Co atoms are slightly different from the pristine bilayer. The average value is slightly shifted towards the Fermi level when H is adsorbed on the Co atom while it is shifted away from the Fermi level when H is adsorbed on the Fe atom. It can be seen that the d band spin splitting is increased when H atom is adsorbed on the Co atom, which in turn reduces the average value of d band center.

In Fig. 8S we present the DOS calculated for hydrogen atoms attached to Fe (black) and Co (red) terminated Fe/Co bilayers. The Bader charge analysis [45] was carried out to understand the charge transfer between the surfaces and adsorbed H atoms. The Bader charges are calculated for Co-H and Fe-H bonded atoms and also for the nearest neighbors to these atoms. From the Bader charges it can be seen that both Co and Fe atoms that are directly bonded to the H atom loose charge and the H atom gains charge. For the Co terminated layer, the Bader charge calculated for the Fe atoms directly bonded to the H are, 0.112e and 0.164 e, and the positive value indicates that charge is lost. The H atom gains charge as indicated by a Bader value of -0.437 e. For the Fe terminated bilayer, the H atom gains -0.353 e and the Co atoms directly bonded to it loose charge as indicated by the Bader values, of 0.088 e and 0.113 e. A closer look at this charge redistribution reveals that the charge transfer is stronger when H atom is attached to the Co atom. This charge transfer in turn affects the magnetic properties and as a result, the magnetic anisotropy energy is different for Fe and Co terminated surfaces.

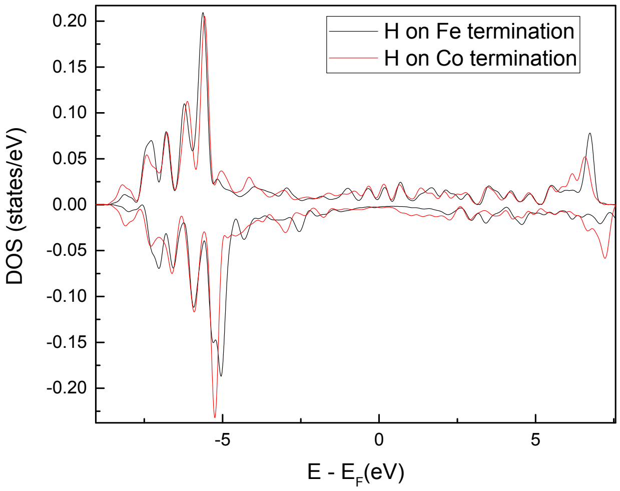

Fig. 8S. The DOS calculated for hydrogen atom attached to Fe (black) and Co (red) terminated Fe/Co bilayers.
